# Vitamin C and Vitamin D—friends or foes in modulating γδ T-cell differentiation?

**DOI:** 10.1038/s41423-022-00895-w

**Published:** 2022-07-07

**Authors:** Christian Peters, Katharina Klein, Dieter Kabelitz

**Affiliations:** Institute of Immunology, Christian-Albrechts University, Kiel, Germany

**Keywords:** Immunology, Cancer

**Fig. 1:**
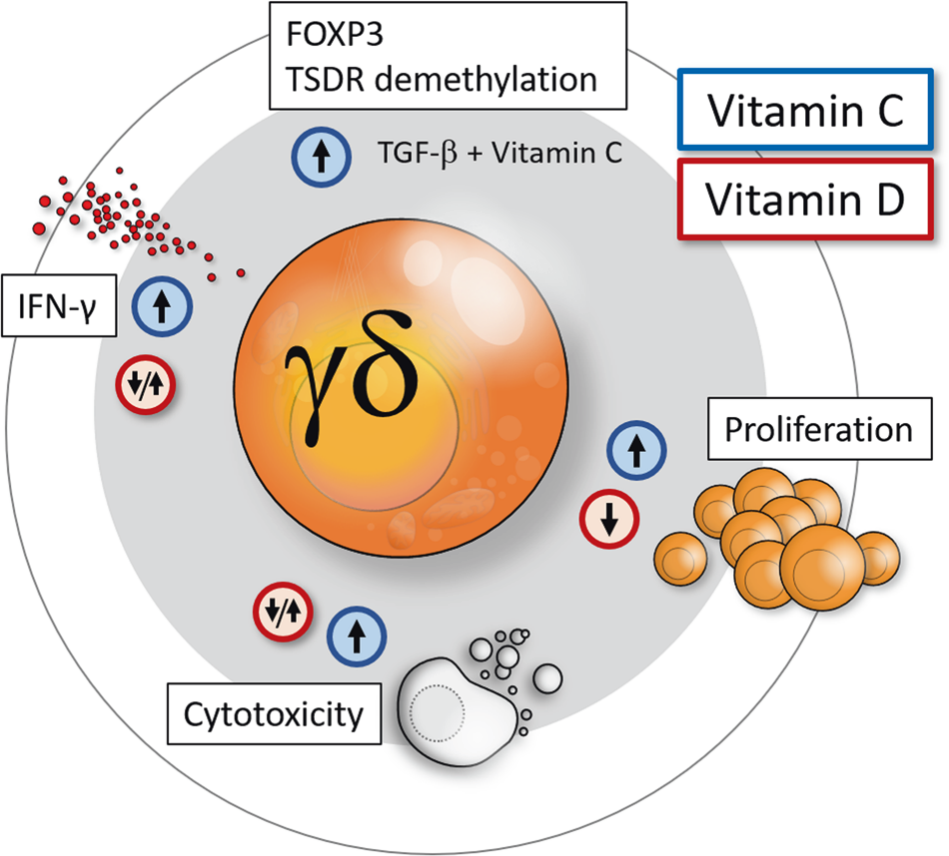
Modulation of human γδ T-cell activation by vitamin C and vitamin D. Vitamin C enhances the cytokine production (notably IFN-γ), proliferative expansion and cytotoxic activity of phosphoantigen-reactive human γδ T cells. In the additional presence of TGF-β, vitamin C induces hypomethylation of TSDRs in the *FOXP3* locus and stabilizes FOXP3 expression in γδ T cells [5–7]. Vitamin D also modulates the IFN-γ secretion, proliferation and cytotoxic activity of human γδ T cells, but the reported effects are controversial. We and others have observed inhibition of proliferation, IFN-γ production and cytotoxicity [13, 14], in contrast to a recent report where the effector functions of γδ T cells were found to be enhanced by vitamin D [15]

The differentiation and functional plasticity of T cells are transcriptionally and epigenetically regulated by signals imposed by the local cytokine milieu and a variety of additional factors, including vitamins. Vitamin C has pleiotropic functions in the immune system. It exerts antioxidant activity, can directly kill selected tumor targets, promotes early T-cell differentiation, and enhances Th1 cytokine production in mature T cells [1, 2]. Vitamin C is also an epigenetic modifier that acts on ten-eleven translocation (TET) enzymes to demethylate *FOXP3* and stabilize FOXP3 protein expression and regulatory T-cell (Treg) function [3]. We previously explored the role of vitamin C in the modulation of the activation and differentiation of human γδ T cells. γδ T cells have recently attracted substantial interest as effector cells in cell-based cancer immunotherapy due to their potent capacity to kill a variety of different cancer cell types in the absence of HLA restriction. While the available data are promising, there is a clear need to optimize the efficacy of γδ T cells in clinical applications [4]. For many potential strategies, including the use of γδ T-cell-selective activating antibodies or γδ T-cell-targeting bispecific antibody constructs and the development of drug-resistant γδ T cells, we reasoned that vitamin C might also be considered to boost the effector functions of γδ T cells Fig. 1.

In a previous study published in this journal, we reported that L-ascorbic acid-2-phosphate (“phospho-vitamin C”, pVC), which is more stable than unmodified vitamin C and does not acidify the cell culture medium, enhances the proliferative expansion and cytokine production of human γδ T cells activated by γδ T-cell-selective pyrophosphates (“phosphoantigens”). Importantly, pVC reduced the proportion of apoptotic cells during the in vitro expansion of γδ T cells. pVC also strongly promoted the cellular expansion of surviving γδ T cells after T-cell receptor restimulation of short-term expanded γδ T-cell lines [5]. When expanded in the presence of pVC, γδ T cells also displayed enhanced metabolic activity and increased killing capacity as measured against several tumor cell targets [5, 6]. Our discovery of the enhancing effect of pVC on the effector functions of γδ T cells led to the first adoptive transfer of allogeneic γδ T cells expanded in vitro in the presence of vitamin C into patients with solid cancers [6].

In view of the known effect of vitamin C on the activation of TET enzymes and thus on the demethylation in Treg-specific demethylated regions (TSDRs) of the *FOXP3* locus and on Treg function, we more recently extended our studies to FOXP3 regulation in human γδ T cells. We observed that pVC actually increased FOXP3 expression in γδ T cells and induced demethylation in *FOXP3* TSDRs but only in the presence of TGF-β, which is involved in Treg induction. In the absence of pVC but presence of TGF-β, cell sorter-purified FOXP3-expressing γδ T cells did not show any hypomethylation in *FOXP3* TSDRs [7]. Taken together, our previous work established that vitamin C and pVC have strong potential to enhance the effector functions of γδ T cells that could likely be extended to other antitumor effector cells such as CAR T cells or NK cells [8]. Importantly, however, the presence of additional factors such as TGF-β (which is frequently encountered in the local tumor microenvironment) could deviate the desired effects of γδ T cells into tumor-promoting effects, e.g., by inducing an “active” Treg phenotype.

In addition to vitamin C, vitamin D is another vitamin with many immunomodulatory effects. Vitamin D plays an important role in bone mineralization and calcium homeostasis. The active form 1α,25-dihydroxyvitamin D_3_ (1,25(OH)_2_D_3_) binds to the nuclear vitamin D receptor (VDR), which acts as a transcription factor regulating a variety of target genes. There is growing evidence that vitamin D exerts a protective role against several types of cancer[9]. Similar to vitamin C, 1,25(OH)_2_D_3_ has also been found to impact T-cell differentiation at multiple levels. In human T cells, it induces FOXP3 protein expression and a suppressive phenotype but – in contrast to vitamin C – does not induce demethylation of TSDRs of the *FOXP3* locus [10]. Moreover, 1,25(OH)_2_D_3_ suppresses interferon-γ (IFN-γ) and promotes interleukin-10 (IL-10) production and thus downregulates T-cell-mediated inflammation [11]. In support of this finding, clinical studies indicate that oral vitamin D supplementation has beneficial effects in patients with autoimmune diseases such as multiple sclerosis [12].

To date, the available information on the potential modulation of γδ T-cell activation by 1,25(OH)_2_D_3_ is limited. It has been reported that 1,25(OH)_2_D_3_ suppresses the in vitro proliferation and IFN-γ production of human phosphoantigen-reactive γδ T cells [13]. Similarly, we also observed reduced production of IFN-γ by γδ T cells in the presence of 1,25(OH)_2_D_3_ [14]. Moreover, we found that 1,25(OH)_2_D_3_ also inhibited γδ T-cell expansion when γδ T cells within PBMCs were stimulated with phosphoantigen or aminobisphosphonate zoledronic acid. In these experiments, the presence of monocytes within PBMCs played a significant role. Furthermore, 1,25(OH)_2_D_3_ also reduced the killing of selected tumor target cells by expanded γδ T cells. Overall, our and previous studies indicate that 1,25(OH)_2_D_3_ downregulates γδ T-cell function [13, 14]. In striking contrast, a recent report by Li et al. actually demonstrated costimulatory activity of 1,25(OH)_2_D_3_ on the IFN-γ and TNF-α production of human γδ T cells and CD8 T cells activated by anti-CD3/CD28 antibody stimulation [15]. The authors also observed that 1,25(OH)_2_D_3_ increased CD28 expression but reduced PD-1, TIGIT and Tim-3 expression on CD8 T cells and γδ T cells and thus reverted their exhausted phenotype. Furthermore, γδ T cells pretreated with 1,25(OH)_2_D_3_ exerted enhanced antitumor activity in vitro and in vivo upon transfer into immunodeficient mice transplanted with human tumor cells. Importantly, the results also indicated that therapeutic supplementation with calcitriol (1,25(OH)_2_D_3_) can revert the exhausted phenotype of CD8 and γδ T cells in cancer patients and enhance their functional activity [15]. Currently, it is difficult to reconcile the mechanistic basis for the discrepancy between published studies regarding the inhibitory [13, 14] and stimulatory [15] effects of 1,25(OH)_2_D_3_ on IFN-γ production in vitro.

However, there is evidence that both vitamin C and vitamin D might exert beneficial effects in cancer patients [1, 8, 9, 15]. Therefore, how the combination of these two vitamins modulates the immune system should be considered. Specifically, we suggest investigating how the combination of vitamin C and 1,25(OH)_2_D_3_ modulates the activity of immune cells implicated in antitumor defense, including γδ T cells. We recently initiated such experiments and analyzed the regulatory interaction between the two vitamins in modulating the IFN-γ production of human CD4 and γδ T cells. Purified CD4 and γδ T cells were preactivated for 2 h with their respective T-cell receptor ligands (staphylococcal superantigens SEA/B for CD4 T cells, phosphoantigen bromohydrin pyrophosphate [BrHPP] for γδ T cells) to allow responsiveness to 1,25(OH)_2_D_3_ by upregulation of VDR before adding pVC, 1,25(OH)_2_D_3_, or the combination of both. As expected, pVC increased the secretion of IFN-γ by CD4 T cells, while 1,25(OH)_2_D_3_ completely suppressed IFN-γ secretion. Our unpublished results indicate that the suppressive effect of 1,25(OH)_2_D_3_ dominated over the stimulatory effect of pVC, as there was still complete inhibition of IFN-γ secretion in the presence of the combination of 1,25(OH)_2_D_3_ and pVC. In line with our previous reports [5, 14], we also found that pVC enhanced and the reduced IFN-γ secretion induced by 1,25(OH)_2_D_3_ in the supernatants of γδ T cells activated for 7 days with BrHPP. In contrast to the case for CD4 T cells, inhibition by 1,25(OH)_2_D_3_ was not complete, and in fact, inhibition was reversed in the additional presence of pVC.

We aimed to extend the findings of our original paper on the effects of vitamin C published in this journal [5], and our unpublished preliminary work indicates that 1,25(OH)_2_D_3_ overrides the costimulatory effect of pVC by completely shutting down IFN-γ secretion in CD4 T cells but much less stringently in γδ T cells. Obviously, such studies need to be expanded to other cytokines and effector functions. Further detailed in vitro experiments and preclinical studies in appropriate mouse models will be required to delineate if and how the two vitamins can be combined to achieve the best antitumor efficacy.
